# Influenza vaccination of primary healthcare physicians may be associated with vaccination in their patients: a vaccination coverage study

**DOI:** 10.1186/s12875-015-0259-0

**Published:** 2015-03-31

**Authors:** Pere Godoy, Jesús Castilla, José María Mayoral, Vicente Martín, Jenaro Astray, Núria Torner, Diana Toledo, Núria Soldevila, Fernando González-Candelas, Susana García, José Diaz-Borrego, Sonia Tamames, Angela Domínguez, the Working Group for the Survey on Influenza Vaccination in Primary Health Care Professionals

**Affiliations:** Agencia de Salud Pública de Cataluña, Barcelona, Spain; CIBER Epidemiología y Salud Pública (CIBERESP), Barcelona, Spain; Instituto de Investigación Biomédica de Lleida, Universidad de Lleida (IRBLleida), Lleida, Spain; Instituto de Salud Pública de Navarra, Pamplona, Spain; Servicio de Vigilancia de Andalucía, Sevilla, Spain; Instituto de Biomedicina, Universidad de León, León, Spain; Área de Epidemiología, Comunidad de Madrid, Madrid, Spain; Departamento de Salud Pública, Universidad de Barcelona, Barcelona, Spain; Servicio de Epidemiología, Agencia de Salud Pública de Barcelona, Barcelona, Spain; Centro Superior de Investigación en Salud Pública, Universidad de Valencia, Valencia, Spain; Unidad de Investigación, Hospital Galdakao-Usansolo, Guipúzcoa, Spain; Servicio Andaluz de Salud, Sevilla, Spain; Dirección General de Salud Pública, Investigación, Desarrollo e Innovación, Junta de Castilla y León, León, Spain

**Keywords:** Vaccine, Influenza, Physician, Coverage, Elderly

## Abstract

**Background:**

To assess the contribution of physician-related factors, especially their influenza vaccine status, in the vaccination coverage of their patients.

**Methods:**

A study of vaccination coverage was carried out in Spain in 2011–12. The dependent variable (vaccination coverage in patients aged ≥65 years) was obtained from regional records. Information was gathered on the vaccination of physicians through an anonymous web survey. We compared the vaccination coverage of patients with the vaccination of their physicians using the Student *t* test. Associations were determined using a multilevel regression model.

**Results:**

The coverage in patients aged ≥ 65 years was 56.3% and was higher (57.3%) in patients whose physician had been vaccinated than in those whose physician had not (55.2%) (p = 0.008). In the multilevel regression model, vaccination of the physician was associated (p = 0.049) with vaccination of their patients after controlling for the effects of age (p = 0.046), region (p = 0.089), and opinions on the effectiveness of the vaccine (p = 0.013).

**Conclusions:**

Vaccination of physicians together with their opinions on the effectiveness of the vaccine may be a predictor of vaccination coverage in their patients. Further studies are required to confirm this.

**Electronic supplementary material:**

The online version of this article (doi:10.1186/s12875-015-0259-0) contains supplementary material, which is available to authorized users.

## Background

Influenza is a highly-contagious disease that causes a significant disease burden [1] and is estimated to affect 5-15% of the world population annually [2]. Health care workers (HCW) may be exposed to the influenza virus in the workplace and can also act as a source of infection of patients and health authorities therefore recommend annual vaccination [1,3-6]. However, although there is evidence on the effectiveness of influenza vaccination, some Spanish [4-7] and international [8-11] studies show that coverages do not generally exceed 40%.

Influenza vaccination has been shown to be effective in protecting the elderly and reducing morbidity and mortality in both institutionalized and community dwellers [12,13]. Therefore, vaccination is generally recommended in this population group [1].

Studies have shown the importance of physicians recommending vaccination to their community-dwelling patients [14,15]. Likewise, an association has been shown between effective vaccination of physicians and the effectiveness of their recommendations to their patients: physicians who are vaccinated have a greater capacity to effectively counsel their patients [16,17].

A weaker and more controversial association between vaccination of primary care physicians and real vaccination of their patients has also been suggested [18].

Primary care physicians are in direct contact with the population and therefore their views on influenza vaccine and the decision to vaccinate may be determining factors in the vaccination of their patients [17,19,20]. In Spain, influenza vaccination is offered free of charge to groups in which it is indicated, including healthcare workers and persons aged ≥ 65 years, in primary healthcare centers after prescription by the patient’s physician. Vaccination is offered in October and November, in a similar fashion to most European countries [21].

The aim of this study was to assess the contribution of physician-related factors, especially their influenza vaccine status, in the vaccination coverage of their patients aged ≥65 years.

## Methods

We conducted an epidemiological study of the prevalence of influenza vaccination coverage in patients aged ≥ 65 and influenza vaccination of primary care physicians according to the Strengthening the Reporting of Observational Studies in Epidemiology (STROBE) statement (guidelines for reporting observational studies) [22]. Of a total of 1791 primary care centers, we randomly selected 253 in seven Spanish regions (Andalusia, Castile-Leon, Catalonia, Valencia, Madrid, Navarre and Basque Country) in the 2011–12 season. A questionnaire was administered anonymously to physicians working in these centers between March 1 and May 25, 2012, via the internet, to obtain information on the main independent variable (influenza vaccination of primary care physician) and other variables (age, gender, education, opinions and attitudes of primary care physicians to influenza vaccination).

The questionnaire was developed after reviewing the scientific literature on the subject, especially the questionnaire used in the study by Kraut et al. [11]. The questions were adapted to the specific circumstances of the Spanish National Health System and two pilot tests were conducted among medical staff in the researchers’ settings to validate understanding of the questionnaire and its length. The final questionnaire consisted of 23 questions (22 closed and one open). Questions were distributed in three sections: information on the history of chronic disease and vaccination, knowledge of influenza and the influenza vaccine, and sociodemographic information. The questions were structured to appear gradually, spread over a total of six screens. The first screen welcomed the participants and provided general information on the survey. The following four screens contained the survey questions, and the last screen contained a text thanking the participants. Using the tools provided by the web platform, 19 of the 23 questions were compulsory, i.e., they had to be answered in order to access the following question.

### Study subjects

The target population was any physician providing direct patient care in primary healthcare centers. In these centers, influenza vaccination is administered without cost by nurses to all population groups for which it is indicated, including people aged ≥65 years, according to physician prescription. Participating centers were randomly selected from a list of the centers in each region. All physicians in each center who had an email address were initially included. The questionnaire was accessible for a month and an email reminder was sent every 10 days to physicians who had not accessed the questionnaire or had not completed the survey.

### Variables

The following variables were collected: profession, age, and sex. We also collected the presence of contraindications to influenza vaccination in each HCW, influenza vaccination in the 2011–2012 season and information on physicians’ knowledge of and opinions and attitudes to influenza and influenza vaccination. Variables related to knowledge of and attitudes to influenza vaccination were covered by a set of questions evaluated on a Likert scale with 5 categories: totally agree, agree quite a lot, neither agree nor disagree, disagree quite a lot, and totally disagree. Information on the dependent variable (vaccination coverage in patients aged ≥ 65 years treated by physicians who participated in the study) was obtained from regional primary care records and was included as study information associated with each individual physician survey.

### Statistical analysis

The data analysis included physicians providing direct patient care in primary care centers who reported information about their vaccination in the 2011–2012 season and in whom we could recover vaccination uptake in their patients aged ≥ 65 years. Physicians in whom vaccination was contraindicated were excluded.

The researchers responsible for each region facilitated listings of the HCW from each center containing the e mail address patients ascribed to each physician and vaccination coverage in their patients aged ≥ 65 years. The Coordinator Center received all lists and formulated a new list which assigned a number corresponding to each participant. This numbering was used to anonymize listings. The new listing was loaded on the web platform. At the end survey period, a database of completed surveys was extracted and was cross-checked with the anonymized list to identify non-responders. To characterize non-answers, we obtained information from 49.1% of physicians who did not respond to the survey. Physicians who responded to the survey were compared to those who did not according to physicians’ age and sex and vaccination coverage in their patients aged ≥ 65 years.

The answers to questions about knowledge and attitudes were dichotomized in two categories: positive (totally agree, agree quite a lot) and negative (neither agree nor disagree, disagree quite a lot, and totally disagree).

A bivariate comparison using the Chi-square test was made in vaccinated/unvaccinated physicians considering the different sociodemographic variables and the answers to questions about knowledge and attitudes. Vaccination coverage in patients aged ≥ 65 years was compared with the main independent study variables using the Student’s *t* test.

The association between vaccination coverage in patients aged ≥ 65 years and the main independent variable (vaccination of their physicians) was determined using a multilevel regression model with input of variables with a significance of p <0.10. All statistical tests were two-tailed and the α error accepted was 0.05. The analysis was performed using SPSS version 18 (SPSS Inc., Chicago, IL).

### Ethical considerations

All information collected was treated in strict observance of legislation on observational studies. The study protocol was approved by the Ethics and Clinical Research Committee of the Jordi Gol Institute for Research in Primary Care.

## Results

The survey was sent to 2535 primary care physicians, of whom 1292 (51.0%) initiated the survey and 872 completed it (34.4%). Physicians who did not respond to the survey were more frequently aged > 55 years (67.0%, 229/342, p = 0.003), but no statistically-significant differences were observed for sex (p = 0.479) or vaccination coverage of patients aged ≥ 65 years (p = 0.146).

Twenty-five physicians who responded to the survey had contraindications to influenza vaccine and 32 did not have valid information on coverage in patients aged ≥ 65 years. Therefore, 815 physicians were finally analyzed, of which 58.4% (476/815) were female. The main age groups were 45–54 years (44.7%, 364/815) and 55–64 years (26.4%, 215/815). A total of 38.7% of participating physicians (315/815) reported having received specific training, 67.2% (548/815) believed that influenza could be a serious illness, 59.4% (484/815) were concerned about infecting their patients, 87.4% (712/815) believed that the vaccine was effective and 49.8% (406/815) were worried about becoming ill due to influenza (Table 1).

**Table 1:** **Characteristics of primary care physicians who responded to the survey, Spain, 2012**

**Variables**	**%**	**n/N**
Sex:		
Female	58.4	476/815
Male	41.6	339/815
Age (years)		
25 – 34	3.3	27/815
35 – 44	25.6	209/815
45 – 54	44.7	364/815
55 – 64	26.4	215/815
Participates in the influenza sentinel system		
Yes	11.4	93/815
No	88.6	722/815
Training activities on influenza		
Yes	38.7	315/815
No	61.3	500/815
I believe that influenza may be a severe illness		
Yes	67.2	548/815
No	32.8	267/815
I worry about infecting my patients		
Yes	59.4	484/815
No	40.6	331/815
I believe the influenza vaccine is effective		
Yes	87,4	712/815
No	12.6	103/815
I worry about becoming ill due to influenza		
Yes	49.8	406/815
No	50.2	409/815

Influenza vaccination coverage in participating physicians was 55.3% (451/815), and was higher in those aged 45–54 years (58.5%, 213/364) and 55–64 years (55.8%, 120/215), in males (57.8%,196/339), in those who had received prior training on influenza (56.8%, 179/315) and in those who believed that influenza was a serious illness (56.9, 312/548), although the differences were not statistically-significant (Table 2), and was significantly higher in physicians who were worried about infecting their patients (69.6%, 337/484; p <0.001), in those who thought that the vaccine was effective (60.1%, 428/712; p <0.001), and in those concerned about becoming ill due to influenza (77.1%, 313/406; p <0.001) (Table 2).

**Table 2:** **Prevalence of influenza vaccination in primary care physicians according to study variables, Spain, 2012**

**Characteristics of primary care physicians**	**Prevalence of vaccination %**	**n/N**	**p** ^**a**^
**Total**	**55.3**	**451/815**	
Sex:			
Female	53.6	255/476	0.129
Male	57.8	196/339	
Age (years)			
25 – 34	37.0	10/27	0.098
35 – 44	51.7	108/209	
45 – 54	58.5	213/364	
55 – 64	55.8	120/215	
Participates in the influenza sentinel system			
Yes	54.8	51/93	0.918
No	55.4	400/722	
Training activities on influenza			
Yes	56.8	179/315	0.498
No	54.4	272/500	
I believe that influenza may be a severe illness			
Yes	56.9	312/548	0.189
No	52.1	139/267	
I worry about infecting my patients			
Yes	69.6	337/484	<0.001
No	34.4	114/331	
I believe the influenza vaccine is effective			
Yes	60.1	428/712	<0.001
No	22.3	23/103	
I worry about becoming ill due to influenza			
Yes	77.1	313/406	<0.001
No	33.7	138/409	

^a^p value.

The mean coverage of patients aged ≥ 65 years ascribed to participating physicians was 56.3% (range 10.1% to 92.5%). The coverage was higher in patients whose physicians had received influenza vaccination (57.3%) than in those whose physicians had not (55.2%) (p = 0.008). Coverages were also higher in patients whose physician was aged < 55 years (56.7%) than in those whose physician was aged ≥ 55 years (55.2%) (p = 0.067). With respect to physicians’ attitudes, opinions and knowledge, the only variable associated with increased vaccination coverage in their patients was considering that influenza vaccination was effective (56.8%) compared with patients whose physician felt that it was not (53.5%) (p <0.005) (Table 3).

**Table 3:** **Influenza vaccination coverage in patients aged ≥ 65 years according to the characteristics of their primary care physician, Spain, 2012**

**Characteristics of primary care physicians**	**Mean vaccination coverage in patients aged ≥ 64 years**	**95% Confidence intervals**	**P** ^**a**^
**Total**	**56.3**	**55.6 – 57.1**	
Influenza vaccination			
Yes	57.3	56.2 - 58.3	0.008
No	55.2	54.1 - 56.2	
Sex:			
Female	56.6	55.7 - 57.5	0.448
Male	56.0	54.7 - 57.2	
Age (years)			
25 – 34	57.3	53.6 - 61.0	0.330
35 – 44	56.7	55.1 - 58.2	
45 – 54	56.8	55.7 - 57.8	
55 – 64	55.2	53.6 - 56.8	
Participates in the influenza sentinel system			0.585
Yes	55.8	53.9 - 57.6	
No	56.4	55.6 - 57.2	
Training activities on influenza			0.199
Yes	57.0	55.9 - 58.0	
No	56.0	54.9 - 57.0	
I believe that influenza may be a severe illness			
Yes	56.5	55.9 - 57.4	0.639
No	56.1	54.7 - 57.5	
I worry about infecting my patients			
Yes	56.0	55.1 – 57.0	0.321
No	56.8	55.5 – 58.0	
I believe the influenza vaccine is effective			
Yes	56.8	56.0 – 57.5	0.005
No	53.5	51.2 – 55.9	
I worry about becoming ill due to influenza			
Yes	54.8	54.0 – 55.7	0.912
No	54.7	53.9 – 55.5	

^a^p value.

In the multilevel regression analysis, a physician’s history of receiving influenza vaccination was associated with the vaccination of their patients aged ≥ 65 years (p = 0.049), after controlling for the effect of age (p = 0.046), region (p = 0.089), opinions on the effectiveness of the vaccine (p = 0.013), concern about infecting their patients (p = 0.071) and concern about becoming ill from influenza (p = 0.652) (Table 4).

**Table 4:** **Factors in primary care physicians associated with influenza vaccination of their patients aged ≥ 65 years in the multilevel regression model, Spain, 2012**

**Characteristics of primary care physicians**	**Regression coefficient β**	**Regression coefficient β 95% CI** ^**a**^		**t** ^**b**^	**p** ^**c**^
**Fixed effects**					
Constant	53.526	49.828	57.225	30.215	0.000
Influenza vaccination (1.Yes; 0.No)	1.650	.005	3.295	1.969	.049
Age (1. 55+; 0. <55 years)	1.675	.031	3.319	2.000	.046
I believe the influenza vaccine is effective (1.Yes; 0.No)	2.866	.596	5.136	2.478	.013
I worry about infecting my patients (1.Yes; 0.No)	−1.579	−3.292	.134	−1.809	.071
I worry about becoming ill due to influenza (1.Yes; 0.No)	-.401	−2.142	1.341	-.451	.652
**Random effects**					
Spanish regions^d^, Estimated variance	11.829 ± 6.952 (p = 0.089)				

^a^Confidence interval.

^b^t value.

^c^p value.

^d^Spanish regions: Andalusia, Castile-Leon, Catalonia, Valencia, Madrid, Navarre and Basque Country.

## Discussion

Influenza vaccination coverage in patients aged ≥ 65 years varied widely, but was within the ranges found by other studies in Spain [23,24] and Europe [8,21], and was associated with the vaccination of their primary care physician, after controlling for the possible effects of physicians knowledge, opinions and attitudes towards influenza. Likewise, vaccination of primary care physicians (55.3%) was higher than that found in other Spanish [4-7] and international [8-10,21] studies.

Both vaccine coverages were far from the European targets for HCW and the elderly [21] and may have been overestimated due to a possible bias caused by a greater response to the survey from physicians with a better vaccination record. We compared the characteristics of early and late responders to the survey as a proxy for non-responders: no differences were found according to age and sex, but influenza vaccine coverage in patients aged ≥ 65 years was higher for physicians who were early responders (Additional file 1: Table S1).

The vaccine influenza coverage in patients aged ≥ 65 years was quite similar to the figures of the Spanish Health Ministry [21], but the coverage in regions participating in the study was slightly higher than in the remaining Spanish regions (Additional file 1: Table S2) [25].

The highest reported European vaccination coverages in patients aged ≥ 65 years are in the Netherlands and some parts of the UK (England, Northern Ireland and Scotland), which reached or almost reached the EU 2014/15 target. Five countries (France, Germany, Ireland, Italy and Spain) reported vaccination coverages of around 60% for this specific age group. Denmark, Finland, Luxembourg, Malta, Norway, Portugal and Sweden reported vaccination coverages of around 50%. The remaining countries were below 50% [21].

The study had some other limitations. The response rate of primary care physicians was low, although similar to that of other studies conducted using web-based questionnaires [11,26-28]. As the questionnaire was self-reported, it was not possible to validate the questions or use interviewers to clarify any disputed points. However, the questionnaire was adapted to the Spanish health system according to a questionnaire used in another study [11]. In addition, a pilot study was carried out. Information on vaccination coverage was collected from the records of healthcare providers for all patients ascribed to each physician in the form of clusters and, therefore, the estimate was not controlled according to the individual variables of each vaccinated patient.

The results of the study provide some relevant information. The effect of vaccination of the primary care physician was a minor determinant of the vaccination coverage of their patients aged ≥ 65 years (57.3% versus 55.2%), but remained statistically-significant after controlling for other potential effects associated with the physician’s knowledge, attitudes and beliefs on influenza vaccination. The 2% increase in coverage, although moderate, should be considered positively, as it relates to physicians in direct contact with the majority of the population [10]. In addition, the advantage conferred by being associated with an intervention (vaccination of physicians) that may reduce the transmission of influenza to vulnerable patients [8] and possibly increase the physician’s confidence in providing counseling on vaccination, should be taken into account [16]. Some studies suggest that medical advice has greater efficacy if it comes from vaccinated physicians [16,29].

Vaccination of patients aged ≥ 65 years was also associated in the multilevel regression model with physicians having a favorable opinion of vaccine effectiveness. This variable has been associated with improved vaccination coverage in other studies [11,30-34] and should be taken into account in educational programs aimed at primary care physicians whose objective is to improve vaccination coverages. However, vaccination of patients was not associated with a higher level of knowledge of their physicians provided by training activities. Therefore, any intervention programs aimed at Spanish primary care Spain should be directed towards improving opinions and attitudes about vaccination rather than trying to increase knowledge.

Interventions to increase influenza vaccination rates in HCW have shown small effects on vaccination behavior, and their long-term success is unknown. Kok et al. [35] suggested that a systematic approach (i.e. intervention mapping) is needed for the successful development and implementation of programs to promote influenza vaccination in HCW, identifying sociocognitive variables that drive the recommended behavior. Other studies show that having a mandatory vaccination policy is the strongest predictor of vaccine coverages in HCW and that implementing such a policy should be a priority for all healthcare agencies [36-38]. In the absence of or in conjunction with a mandatory vaccination policy, other interventions may be implemented to increase vaccination compliance in HCW. These include reducing barriers to vaccination, encouraging staff to be vaccinated, and introducing educational campaigns [36,39], all of which suggest the need for healthcare administrators to be active in encouraging vaccination in HCW. Healthcare agencies should provide free vaccination on site to their staff whenever possible to increase compliance [36,39]. This is even more critical in nonhospital settings. Educational campaigns based on beliefs aligned with scientific evidence and more favorable attitudes toward vaccines can also improve the intent of HCW to be vaccinated.

There is sufficient evidence that increases in the vaccination of people aged ≥ 65 years leads to a reduction in mortality and morbidity in both institutionalized [13,40] and community-dwelling [12,40] patients. In addition, clinical trials show it is feasible to increase the vaccination of primary care physicians. A clinical trial by Abramson et al. [18] found that vaccination coverage in the intervention group was 52.8% compared with 26.5% in the control group. However, unlike in our study, these authors did not show that this increase in the vaccination of physicians led to increased vaccination of their patients.

## Conclusions

Our results show that vaccination of primary care physicians was a minor determinant of the vaccination coverage of their patients aged ≥ 65 years, but remained statistically-significant after controlling for other potential effects associated with the physician’s knowledge, attitudes and beliefs on influenza vaccination. Moreover, influenza vaccination was also associated with favorable opinions about vaccination. Therefore, the promotion of influenza vaccination of primary care physicians through improving their opinions and attitudes about influenza vaccination may have a beneficial effect on vaccination coverages in their patients. Interventional studies to increase influenza vaccination of primary care physicians in order to determine whether this leads to increased vaccination coverages in their patients aged ≥ 65 years are warranted.

## Additional file

Additional file 1:
**Table S1.** Characteristics of early and late responders among primary healthcare physicians and influenza vaccine coverage in their patients aged ≥65 years. **Table S2.** Influenza vaccine coverage in persons aged ≥ 65 years in Spanish regions participating or not in the study.
