# Supplementary material for: Influenza vaccination of primary healthcare physicians may be associated with vaccination in their patients: a vaccination coverage study
Source: BMC Fam Pract. 2015 Mar 31;16:44. doi: 10.1186/s12875-015-0259-0 (PMC4389995; doi:10.1186/s12875-015-0259-0)
Supplement: Additional file 1: — Table S1. Characteristics of early and late responders among primary healthcare physicians and influenza vaccine coverage in their patients aged ≥65 years. Table S2. Influenza vaccine coverage in persons aged ≥ 65 years in Spanish regions participating or not in the study. [file 12875_2015_259_MOESM1_ESM.doc]

**Additional file**

**Table S1. Characteristics of early and late responders among primary healthcare physicians and influenza vaccine coverage in their patients aged ≥65 years**

| **Characteristics of primary care physicians** | **Before 2^nd^ reminder** | **After 2^nd^ reminder** | **P** |
| --- | --- | --- | --- |
| Sex:  Female  Male | 289 (60.7%)  191 (56.3%) | 187 (39.3%)  148 (43.7%) | 0.21 |
| Age (years)  25 – 34  35 – 44  45 – 54  55 – 64 | 16 (59.3%)  129 (61.7%)  212 (58.2)  123 (57.2%) | 11 (40.7%)  80 (38.3%)  152 (41.8%)  92 (42.8%) | 0.799 |
| Coverage in patients aged ≥65 years | 57.7±11.2 | 54.4±10.3 | <0.001 |

**Table S2. Influenza vaccine coverage in persons aged ≥ 65 years in Spanish regions participating or not in the study**

| **Region** | **Population** | **Vaccinated** | **%** |
| --- | --- | --- | --- |
| Study regions ^a^ | 6,001,777 | 3,439,405 | 57.3 |
| Other regions^b^ | 2,575,289 | 1,397,908 | 54.4 |
| TOTAL | 8,577,066 | 4,837,313 | 56.4 |

^a^ Spanish regions: Andalusia, Castile-Leon, Catalonia, Valencia, Madrid, Navarre and Basque Country

b Other regions: Aragon, Asturias, Balearic Islands, Canary Islands, Cantabria, Castile La Mancha, Extremadura, Galicia, Murcia, La Rioja, Ceuta, Melilla

Source: <https://www.msssi.gob.es/profesionales/saludPublica/prevPromocion/vacunaciones/coberturas.htm>
